# Photo-catalytic and biological applications of phyto-functionalized zinc oxide nanoparticles synthesized using a polar extract of *Equisetum diffusum* D

**DOI:** 10.1039/d4ra03573a

**Published:** 2024-07-15

**Authors:** Nasir Assad, Azhar Abbas, Muhammad Fayyaz ur Rehman, Muhammad Naeem-ul-Hassan

**Affiliations:** a Institute of Chemistry, University of Sargodha Sargodha 40100 Pakistan sobheel@yahoo.com +923338967518; b Department of Chemistry, Government Ambala Muslim College Sargodha 40100 Pakistan

## Abstract

In this study, zinc oxide nanoparticles (ZnO NPs) were fabricated using *Equisetum diffusum* D extract and their diverse properties and applications were studied. Phytochemical analysis confirmed the presence of phenols and flavonoids in the plant extract, playing a crucial role in the stabilization and reduction of the synthesized nanoparticles. The greenly synthesized ZnO NPs were characterized through a range of analytical techniques. UV-visible spectrophotometry has been employed to investigate their optical characteristics. FTIR spectroscopy was employed to identify the functional groups responsible for the synthesis of the ZnO NPs. The structural properties were evaluated using XRD. The morphology and size distribution of the synthesized NPs were examined using SEM, DLS, and elemental spectra evaluated using EDX. The charge that develops at the interface was analyzed using zeta potential which accounts for stability of the NPs. The ZnO NPs exhibited excellent photocatalytic degradation of cationic (methylene blue), anionic (methyl orange), and nonionic (*p*-nitrophenol) dyes under sunlight exposure with photocatalytic degradation of 85.61%, 79.10%, and 89.95% respectively. Additionally, the nanoparticles displayed antimicrobial activity against Gram-positive and Gram-negative bacteria, and noteworthy antioxidant potential. The anti-inflammatory activity of the ZnO NPs, attributed to their ability to inhibit protein denaturation, was dose-dependent. Overall, our findings highlight the versatile properties of the greenly synthesized ZnO NPs, showcasing their potential in environmental remediation, and antimicrobial formulations, and as promising candidates for further exploration in the biomedical fields, including drug delivery and therapeutics.

## Abstract 2

In this study, zinc oxide nanoparticles (ZnO NPs) were fabricated using *Equisetum diffusum* D extract and their diverse properties and applications were studied.

## Graphical abstract

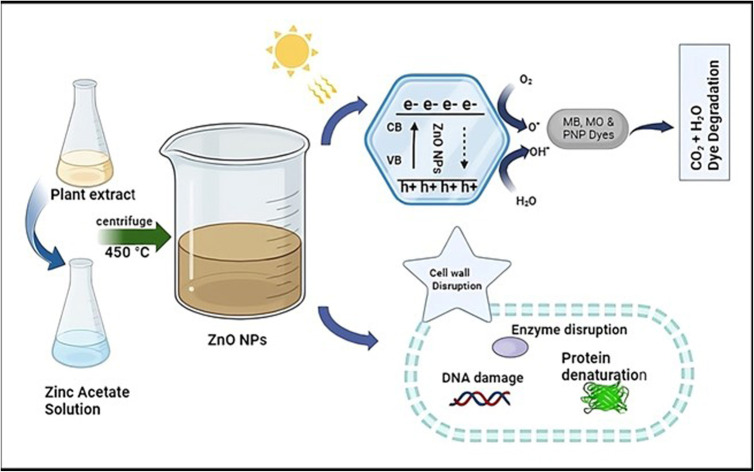

## 1 Introduction

Cationic and anionic dyes are a common category of organic pollutants.^1^ Clothing, paper industry, suede, nutrition, printing purposes, and medical fields are all dependent heavily on organic dyes, making them an essential component in the industrial sector. Pigmentation for many materials accounts for the consumption of about 60% of dyes in the textile industry.^2^ After the fabric process, approximately 15% of the dyes are discarded and released into the hydrosphere processing. By their refractory nature, these dyes constitute a significant source of pollution.^3^ The most significant contributors to ecological pollution are the by-products of these industries.^4^ MB is a cationic azo dye. It's often used to color silk, paper, hemp, and wood,^5^ and as a chemical ingredient in the printing and dying industries.^6^ MB dye causes vomiting, anxiety, respiratory issues, and hyperhidrosis. Textile and leather manufacturers depend heavily on MO dye for their coloring purposes. The release of MO poses major risks to the environment and can cause cancer and mutations. Dye concentrations as low as 1.0 mg L^−1^ produce thick shading on water that is both harmful to aquatic life and unfit for human consumption.^7^*P*-Nitrophenols (PNPs) are introduced to the soil and water supply by the agricultural sector *via* the widespread use of organic phosphorus insecticides. Furthermore, PNPs pose a significant risk to aquatic and human life because they are persistently poisonous and carcinogenic.^8,9^ PNP is one of the most harmful pollutants, according to the United States Environmental Protection Agency (EPA), with a permissible limit of 1–20 mg L^−1^ for commercial sewage.^10^

The tannery effluent discharged into wastewater streams and soil, has a potential to generate significant environmental complications due to its carcinogenic and extremely toxic characteristics. These effluents are directly released into water streams without pretreatment.^11^ Thus, to ensure the environment's sustainability for future generations, it is essential to develop efficient treatment strategies for the organic dyes in wastewater. In comparison to more conventional approaches to water treatment, like biological processes, flocculation, and chemical treatment, photooxidation has been found to be a more efficient, cost-effective, and ecologically benign way to degrade the dyes and remediate wastewater.^12,13^

An exciting new development in this area is semiconductor photocatalysis, which uses nanomaterials to destroy a wide range of contaminants at room temperature and pressure. This highlights the need to research and develop photocatalytic materials with enhanced efficiency for oxidative degradation.^14^ Recently there has been a lot of research into using transistors, like TiO_2_, ZnO, WO_3_, and ZrO_2_ as photocatalysts to degrade different types of pigments.^15,16^ Among these, ZnO and TiO_2_ are becoming popular in solar cells, photocatalytic systems, and light-emitting devices.^17,18^ On a larger scale, though, ZnO is more cost-effective than TiO_2_, making it favourable on the commercial scale.^19^

ZnO NPs have gained a lot of attention due to their promising photocatalytic properties, lack of toxicity, large band gap, photosensitivity, high binding energy, and exceptional room temperature chemical stability.^20^ ZnO NPs have found several uses in various fields, including catalysis, antioxidant activity, environmental protection, electronics, cosmetics, communication, biology, and pharmaceutical industries.^21–23^ Furthermore, due to their antimicrobial,^24^ antifungal,^25^ anti-diabetic,^26^ and insecticidal^27^ features, ZnO NPs have a vast variety of medical utilities that include bio-sensing,^28^ drug transport,^29^ and nanomedicines.^30^ The use of different parts of plant extracts is a promising technique for the synthesis of ZnO NPs. Sivasankarapillai *et al.*, (2023), demonstrated a simple and eco-friendly method for synthesizing ZnO NPs from Scoparia Dulcis extract.^31^ Seghir *et al.*, (2023), explored the antimicrobial potential of green-synthesized ZnO NPs from *Zingiber officinale* and *Glycyrrhiza* root extracts.^32^ Chandrasekaran *et al.*, (2023), synthesized ZnO NPs by using water-based floral extract of Senna auriculata as a reducing agent and assessment of their antibacterial and antidiabetic potential.^33^

In this study, *Equisetum diffusum* D plant polar was used as a reducing and stabilizing agent for the biogenic synthesis of ZnO NPs. The greenly synthesized ZnO NPs were characterized using a range of analytical techniques. UV-vis and FTIR spectroscopy, X-ray diffraction, scanning electron microscopy, energy dispersive X-ray spectroscopy, dynamic light scattering, and zeta potential analyses provided a comprehensive understanding of the nanoparticles' structure, morphology, and stability. These ZnO NPs were assessed for their potential for photocatalytic efficacy by subjecting methylene blue (MB), orange (MO), and *p*-nitrophenol (PNP) as representative dye molecules under diffused solar irradiation. Additionally, we performed a comprehensive investigation for the antibacterial potential for both Gram-negative and Gram-positive bacteria, together with the antioxidant activity using various methods, such as DPPH, TPC, TFC, and FRAP, activity. As an outcome, the unique catalytic and optical features of synthesized ZnO NPs, along with the reducing and stabilizing potential of plant extract, make these NPs a cost-effective and simple candidate for potential use in health and industrial applications.

## 2 Materials and methods

### 2.1 Materials

*Equisetum diffusum* D herbs were collected from North Waziristan Tribal district, Khyber Pakhtunkhwa, Pakistan. A botanist from the Department of Botany, University of Sargodha, Sargodha, Pakistan, identified and authenticated the plant stem of *E. diffusum* D. Zinc acetate (Merck, Germany), DPPH (Sigma-Aldrich®), *n*-hexane (EMSURE ACS, Malaysia) and ethanol (Sigma-Aldrich®) were bought from the local marketplace and were of analytical grade. Deionized water (DH_2_O) was utilized for solution preparation and extractions.

### 2.2 Preparation of extract

The freshly collected stems of *E. diffusum* D were cleaned in both tap water and distilled water to remove any traces of dust. The *E. diffusum* D stems were cut into tiny pieces and dried under the shade for 15 days following the protocol of Assad *et al.*, (2023) with a few modifications.^34^ The fragments were pulverized to a fine powder by using a pestle and mortar. Plant powder suspension was prepared by taking 5 g of fine plant powder in 100 mL DW and was agitated at ambient temperature. After that, the mixture was filtered through Whatman filter paper no. 42, followed by washing the filtrate with *n*-hexane using a separating funnel and after washing the layers *i.e.* polar and non-polar were separated. The non-polar layer was washed out, however, the polar layer was discharged into a Petri dish and then dried for 24 h in an air-drying oven at 45 °C. When the polar extract was completely dried, it was retrieved from the Petri dish, carefully packed in an Eppendorf tube and stored in the refrigerator till further use.

### 2.3 Phytochemical analysis

Phytochemical analysis was performed on *E. diffusum* D polar extract in accordance with the previously published protocol by Silva *et al.*, (2017). The following tests were employed to recognize the existence of various compounds, including alkaloids (Wagner test), flavonoids (lead acetate test), glycosides (sodium hydroxide test), lignins (labat test), saponins (foam test), coumarins (sodium hydroxide test), phenols (ferric chloride test), sterols (Salkowiski test) leucoanthocyanin (isoamyl alcohol test), and fatty acids (Mojonnier method).^35^

### 2.4 Synthesis of nanoparticles

ZnO NPs were synthesized applying a protocol by Ghaffar *et al.*, (2023) with minor alterations.^14^ In a 250 mL volumetric flask, 0.68 g 100 mL^−1^ zinc acetate aqueous solution was added drop-wise to 50 mL of *E. diffusum* D stem extract under continuous and vehement stirring for 30 min. The solution was then heated to 70 °C till a pale yellow-coloured precipitate was formed. This pale yellow precipitate was centrifuged at 6000 rpm for 20 min, rinsed with DH_2_O two to three times, and finally calcined at 450 °C. The dried ZnO NPs were scraped, packed, labelled as ZnO@ED NPs and stored till further analysis.

### 2.5 Characterization of ZnO@ED NPs

The UV-vis absorption spectrum of biosynthesized ZnO@ED NPs was recorded by a UV-visible spectrophotometer (Shimadzu UV-1800) at 300 to 600 nm wavelength range, functional group starching and functional groups of plant molecules present on particles surface, Fourier transform infrared (FTIR) spectra of polar extract and biosynthesized ZnO@ED NPs were evaluated using Shimadzu FTIR 8400S at 4000–500 cm^−1^ range using KBr pellets technique. The phase identity and crystalline size of ZnO@ED NPs were characterized by X-ray diffraction XRD (JDX-3532, JEOL, Tokyo, Japan). Morphological features were studied by scanning electron microscopy (Carbon Sticker No G3347) Plano (Wetzlar, Germany) and elemental spectra of ZnO@ED NPs were also evaluated by EDX. The charges that developed at the interface were analyzed by zeta potential and the size was determined by zeta size (Malvern Zetasizer Nano ZS) distribution analyzer.

### 2.6 Photocatalytic activity of ZnO@ED NPs

The photocatalytic degradation of MB, MO, and PNP by ZnO@ED NPs as catalyst was performed in the sunlight following a protocol from Ghaffar *et al.*, (2023), with a few modifications.^14^ First 30 mL of MB, MO, and PNP dye solutions (10 ppm each) were mixed with 20 mL catalyst (1 mg mL^−1^) and stirred for 30 min in the dark on an orbital shaker until equilibrium absorption of MB, MO, and PNP on the photocatalysts occurred. After stirring, the solutions were subjected to solar radiation for 120 min. At 0, 10, 20, 40, 60, 80, 100 and 120 min, 3 mL of the whole solution was obtained and examined by a UV-visible spectrophotometer to evaluate the rate of the reaction. Time-dependent decreases in the absorbance peaks at 665, 460 and 400 nm for MB, MO and PNP, respectively, indicate that the ZnO@ED NPs synthesized here effectively degraded the dyes through their photocatalytic activity.

### 2.7 Evaluation of antibacterial activity

Four distinct strains of Gram-positive and Gram-negative bacteria were tested for the antibacterial potential of ZnO@ED NPs employing the agar well diffusion technique following the protocols of Khan *et al.*, (2023) and Jabbar *et al.*, (2023) with some amendments.^36,37^ A total of 6.3 g of Mueller Hinton (MH) agar (Oxoid) was solubilized in 120 mL of DH_2_O. The Petri plates and agar solution were autoclaved for 20 min at 121 °C. Following the sterilization process, the medium was cooled down to 50 °C. Subsequently, 25 mL of agar solution was evenly distributed onto each Petri plate and kept there for 20 min to solidify. Pure cultures of the organisms were subcultured on nutritional agar in a rotary shaker set at 200 rpm at 35 °C for 24 h. Then the 24 h mature cultures of *Listeria monocytogenes* (ATCC#13932), *Staphylococcus epidermidis* (ATCC#12228), *Escherichia coli* (ATCC#10536) and *Bordetella bronchiseptica* (ATCC#4617) were inoculated to each Petri plate using the plate streaking method. Using a sterile cork borer, four separate wells were drilled into each Petri dish and the antibacterial potential of the ZnO@ED NPs was evaluated using a sample concentration of 30 μL per well. Alphabetically labelled four wells on each Petri dish were loaded with 30 μL of ciprofloxacin (1 mg mL^−1^) as a positive control, 30 μL ZnO@ED NPs (1 mg mL^−1^), 30 μL plant extract (1 mg mL^−1^) and 30 μL DH_2_O as a negative control. The plates were thereafter put in an oven, adjusted at 37 °C, and left undisturbed for about 24 h. As the incubation time was completed, the sizes of the zones of inhibition in each well were evaluated and documented. The process was tested three times, and the antibacterial activity was estimated by averaging the three results.

The minimum inhibitory concentration (MIC) of green-synthesized ZnO@ED NPs was determined using the standard technique established by the Clinical and Laboratory Standards Institute (CLSI).^38^ Both Gram-positive and Gram-negative strains were used in this investigation to determine MIC. Four strains *Listeria monocytogenes* (ATCC#13932), *Staphylococcus epidermidis* (ATCC#12228), *Escherichia coli* (ATCC#10536) and *Bordetella bronchiseptica* (ATCC#4617) were grown in a shaking incubator at a temperature of 37 °C with an agitation speed of 200 rpm for 24 h, using MH broth medium. The bacterial inoculum concentration was adjusted to 105 CFU mL^−1^. The ZnO@ED NPs were tested for their MIC using the conventional broth microdilution methodology. To determine the MIC, a solution containing the synthesized ZnO@ED NPs was prepared at a concentration of 1 mg mL^−1^. The MIC was then determined by evaluating a range of diluted values, from 200 to 10 μg mL^−1^. There were also negative control experiments with nutrient broth and ZnO@ED NPs (200 μg mL^−1^), as well as positive control experiments with nutrient broth and only bacterial strains. The tubes were thereafter incubated at a temperature of 37 °C for 24 h. After incubation, the concentration of ZnO@ED NPs at which bacterial growth is completely inhibited was determined.

### 2.8 Antioxidant activity

#### 2.8.1 Ferric reducing antioxidant power (FRAP) assay

The FRAP assay was executed in accordance with methodology illustrated by Shobha *et al.*, (2019) with some amendment.^39^ In brief, different concentrations of ZnO@ED NPs solution, in the range of 100 to 500 μg mL^−1^ combined with 2.5 mL of sodium phosphate buffer (PBS) (0.2 M, pH 6.6) and 2.5 mL 1% potassium ferricyanide and incubated for 20 min at 50 °C. Then a 10% w/v solution of trichloroacetic acid was added and the resulting solution was centrifuged for 10 min at 3000 rpm. 2.5 mL of deionized water and 0.1% ferric chloride solution were added to the supernatant. Then absorbance was determined at 700 nm by a spectrophotometer (Shimadzu UV-1800). To determine FRAP′s antioxidant potential, the following eqn (1) was used.^40^1
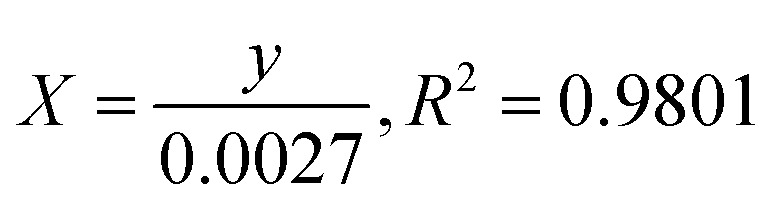


#### 2.8.2 2,2-Diphenyl-1-picrylhydrazyl (DPPH) assay

According to methodology of Khan *et al.*, (2023), various concentrations (from 100 to 500 μg mL^−1^) of ZnO@ED NPs, were combined with 2 mL of methanol and 1 mL of a methanolic solution containing DPPH free radicals (0.1 mM).^36^ Following thorough mixing of the constituents, the solutions were subsequently incubated at ambient temperature for 30 min in dark environment. To determine the absorbance, a spectrophotometer (Shimadzu UV-1800), set at 517 nm was used. To determine FRAP's antioxidant potential, the following eqn (2) was used.^36^

2 (Absorbance of control 517 − absorbance of sample 517)/absorbance of control 517 × 100

### 2.9 Total polyphenol content (TPC)

The Folin–Ciocalteu method was used to estimate the total polyphenol content following the protocol of Noreen *et al.*, (2017), with few changes.^41^ First, 2 mL of DH_2_O and 250 μL of 1 N Folin Ciocalteu's phenol were mixed with 100, 200, 300, 400 and 500 μL of ZnO@ED NPs and placed in the dark for 8 min at room temperature. Thereafter, 750 μL of a 20% Na_2_CO_3_ solution and 950 μL of DH_2_O were added. The solutions were kept in the dark environment for 30 min, then the absorbance was recorded at 765 nm *via* a Shimadzu UV-1800 spectrophotometer. The resulting values were represented as g GAE per mL of ZnO@EDNPs, with gallic acid serving as the reference. The level of scavenging was estimated using the eqn (3).^40^3
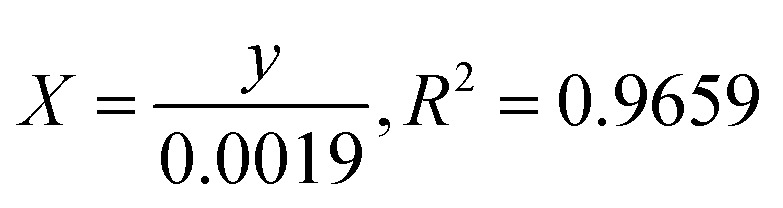


### 2.10 Total flavonoid content (TFC)

Colorimetric method using aluminium chloride (AlCl_3_) was employed to determine the concentration of flavonoid, following the protocol of Sana *et al.*, (2020).^42^ In brief, 0.75 mL of methanol was combined with 100, 200, 300, 400, and 500 μL (1 mg mL^−1^) of ZnO@ED NPs, and the overall volume was increased to 2 mL using DH_2_O. Then, 300 μL of 5% sodium nitrate and 300 μL of 10% AlCl_3_ were added to each sample and then left to settle for 10 min. Further, 2 mL of 1 mol L^−1^ NaOH was added to the solution and the total volume was made up to 5 mL with DH_2_O. The test solution was incubated at room temperature for 40 min before recording the absorbance by spectrophotometer (Shimadzu UV-1800) at 510 nm. The results were determined using quercetin as the reference standard and represented as mg of quercetin equivalents per g (mg quercetin g^−1^) of ZnO@ED NPs. The level of scavenging was calculated using the eqn (4).^40^4
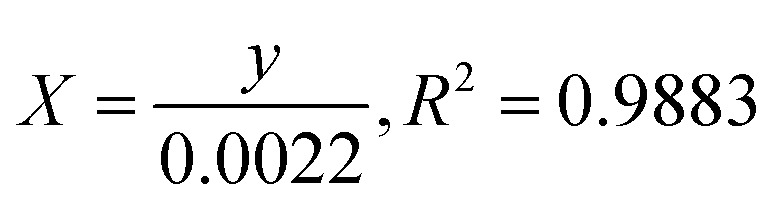


### 2.11 Anti-inflammatory activity

ZnO@ED NPs anti-inflammatory effects were studied by denaturing proteins as per the protocol proposed by Jemaa *et al.*, (2017), with a few minor modifications.^43^ For making a total volume of 5 mL, various concentrations of ZnO@ED NPs (ranging from 0.1 mg mL^−1^ to 0.5 mg mL^−1^) were combined with 2.8 mL of pH 6.4 phosphate-buffered saline (PBS) and 0.2 mL of fresh egg albumin solution. After a 20 min incubation period at 37 °C, the mixtures were subjected to a 5 min heating at 70 °C. Diclofenac sodium (0.1 mg mL^−1^ to 0.5 mg mL^−1^) was used as a standard drug. A control solution was made by combining 2.8 mL of PBS (pH 6.4) with 0.2 mL of egg albumin solution. The volume was increased to 5 mL by adding DH_2_O. To measure turbidity at 660 nm, a UV-vis spectrophotometer (Shimadzu UV-1800) was used. Phosphate buffer was used as a control. We calculated the inhibition of protein denaturation using the eqn (5) given below.^44^

5 Inhibition of protein denaturation (%) = (A660 of sample/A660 of control −1) × 100

## 3 Results and discussion

### 3.1 Phytochemical analysis

The existence of several phyto-constituents was established by phytochemical analysis and the results are shown in Table 1. These results demonstrate that the plant extract contains several important phytochemicals, including phenols and flavonoids that get involved in the biosynthesis of NPs. The role of these chemical groups, as reducing and stabilizing agents, in the green synthesis of ZnO NPs has now been well established. Zinc salts are reduced and stabilized into ZnO NPs through hydroxyl groups found in phenols and flavonoids.^45^

**Table 1:** Phytochemical screening of *E. diffusum* D extract, (+positive, −negative)

Test	Result
Alkaloids	+
Flavonoids	+
Glycosides	−
Lignin	−
Saponins	+
Coumarins	−
Phenols	+
Sterols	+
Leucoanthocyanin	−
Fatty acid	+

### 3.2 UV-vis spectroscopy

**Fig. 1:**
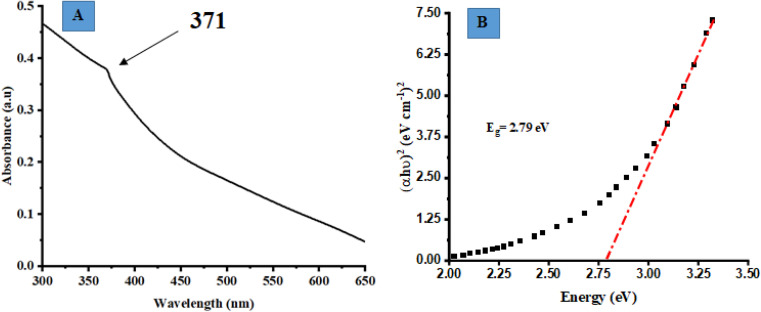
(A) UV-vis absorption spectroscopy of the synthesized ZnO@ED NPs, (B) the optical band gap (*E*_g_) determination of ZnO@ED NPs using the Tauc equation.

UV-vis absorption spectroscopy was employed to analyze the optical characteristics of biosynthesized ZnO@ED NPs. The biosynthesized ZnO@ED NPs exhibited a consistent pale yellow colour throughout the whole synthesis procedure. The absorbance peak, due to the surface plasmon resonance phenomenon was observed at 371 nm, as depicted in Fig. 1(A). The excitation of free electrons is responsible for the appearance of this peak.^45^ ZnO NPs have the potential to be synthesized using environmentally friendly methods, primarily attributed to the bio-reductive and stabilizing properties exhibited by plant metabolites.^46^ The phenomenon of surface plasmon resonance is observed as a chromatic shift, resulting from the conversion of zinc acetate into ZnO NPs (NPs).^14^ The UV-vis analysis was comparable with previous literature.^46,47^ This study provides evidence that the phytochemical components of *Equisetum diffusum* D possess the capacity to function as a bio-reductant, which is very crucial for the photosynthesis of ZnO@ED NPs.^48,49^

The optical band gap (*E*_g_) of the synthesized ZnO@ED NPs particles was determined using the Tauc eqn (6), which was applied to the UV-vis data.^50^

6 (*εhv*)^2^ = *K*(*hν* − *E*_g_)

Eqn (1) represents a relationship between various parameters in the context of direct allowed band gap materials. In this equation, *h* denotes Planck's constant, *K* is an energy-independent constant, *ε* is the molar extinction coefficient, *ν* represents frequency and *n* is a variable dependent on the type of transition. For direct permitted band gap materials, the value of *n* is equal to 2. Fig. 1(B) depicts a graphical representation of the (*h*)^2^ plotted against *h*. The red line on the graph represents an estimation of the average band gap, which is determined by calculating the intercept of the linear component of this plot. The nanoparticles exhibited an energy gap (*E*_g_) of 2.79 eV, which is relatively less than bulk ZnO (3.37 eV). It is shown qualitatively that the band-gap energy decreased as the calcination temperature increased.^51^

### 3.3 X-ray diffractometer (PXRD)

**Fig. 2:**
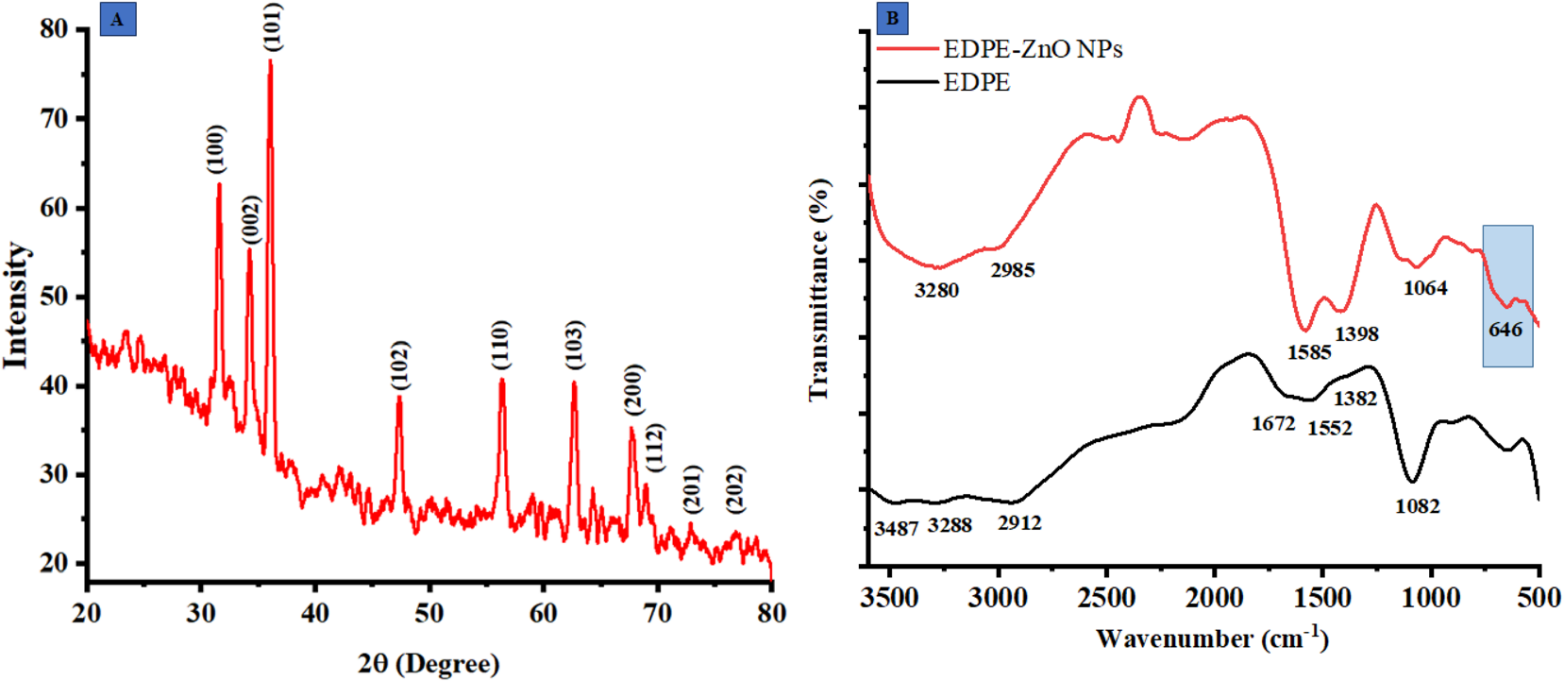
(A) X-ray diffractometer (PXRD) pattern of ZnO@ED NPs, (B) Fourier transform infrared spectroscopy of ZnO@ED NPs and *E. diffusum* D extract.

The X-ray diffraction (XRD) analysis is a non-destructive methodology used for the determination of the chemical composition, physical characteristics, and crystallographic arrangement of crystalline substances. The findings of this study revealed that the extract contained reducing agents that had the capability to synthesize ZnO@ED NPs with enhanced electrical and optical characteristics. The XRD pattern of the ZnO@ED NPs exhibited well-defined diffraction peaks, suggesting the presence of a hexagonal wurtzite crystal structure. The diffraction angles observed were 31.674°, 34.423°, 36.158°, 47.571°, 56.647°, 62.94°, 67.859°, and 69.174°, corresponding to the crystallographic planes (100), (002), (101), (102), (110), (103), (112), and (202), respectively as shown in Fig. 2(A). It was evident from the XRD peaks that there was line broadening, indicating that the produced particles are nanoscale.

The average size of the particles' crystallites was found by the Debye–Scherrer formula (eqn (7)).7
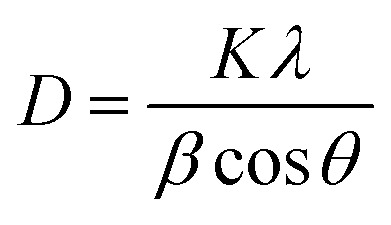


The average size of the particles' crystallites yielded an approximate value of 18.84 nm. The X-ray diffraction pattern reveals prominent peaks at (100), (002), and (101), which are indicative of the wurtzite crystal structure of zinc oxide ^52^. The XDR spectrum of the synthetic ZnO@ED NPs demonstrated a close correspondence between the diffraction peaks and the hexagonal wurtzite structure of ZnO, as specified by the Joint Committee on Powder Diffraction Standards (JCPDS) Card Number 36-1451 and as described previously.^52,53^

### 3.4 Fourier transform infrared spectroscopy (FTIR)

Fourier transform infrared spectroscopy was employed to investigate the function of ZnO@ED NPs. The functional groups in the FTIR spectra of ZnO@ED NPs and plant extract, scanned in the range of 4000–400 cm^−1^, are shown in Fig. 2(B). Several peaks of interest at various wavelengths were observed in the FTIR spectra of ZnO NPs, including the peaks at 3280, 2985 1585, 1398, and 1064 cm^−1^. The OH containing peak at 3487 present in plant extract FTIR spectra, completely disappeared in ZnO NPs spectra of FTIR. The shift change of wide-band spectra associated with OH-containing groups in the spectra of ZnO@ED NPs suggests that the phenolic component of the plant extract serves as a stabilizing and reducing agent.^54^ Prominent stretching at 1382 cm^−1^ in polar extract represented the primary alcohol, shifted to 1398 conforming ZnO NPs.^55^ The presence of the metal oxygen groups is confirmed by the bending vibration peak at 646 cm^−1^, which indicates the establishment of the Zn–O bond in this region.^56^ Our results provide support to the hypothesis that phytochemicals included in *E. diffusum* D polar extract were very crucial in the synthesis of ZnO NPs by facilitating electron transfer and serving as reducing and stabilizing agents.

#### 3.5 Scanning electron microscopy (SEM)

**Fig. 3:**
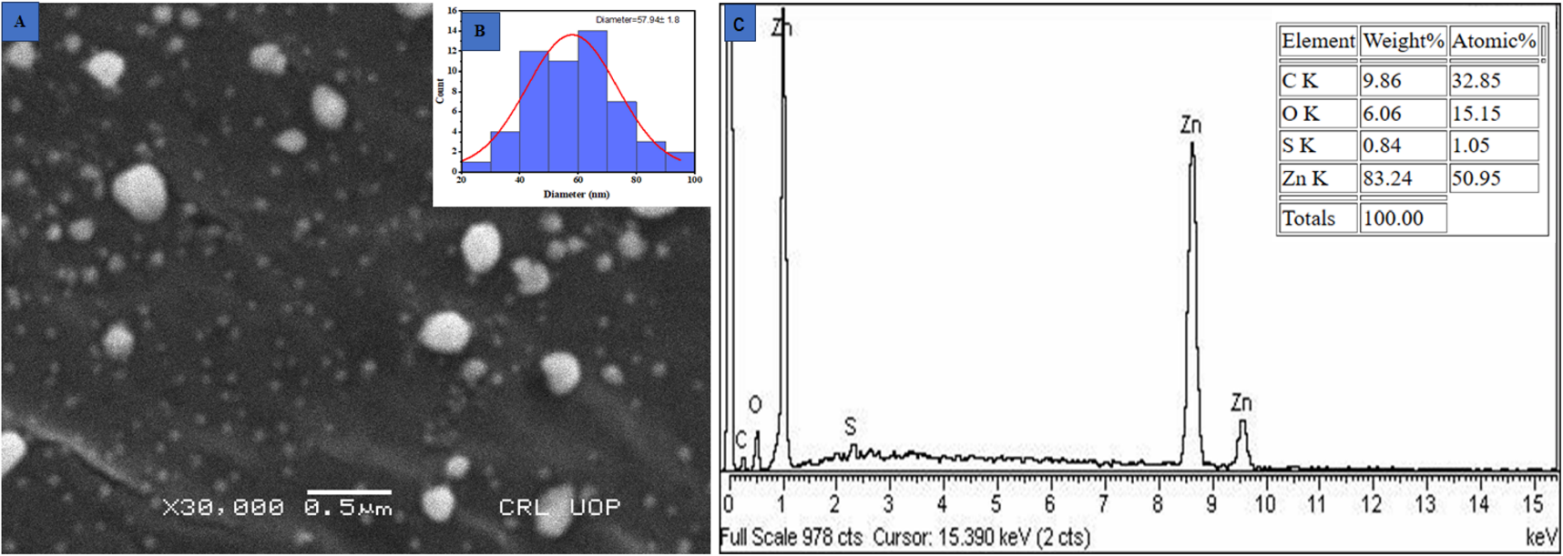
(A) Scanning electron microscope image, (B) histogram of particle size distribution and (C) EDX spectra of the greenly synthesized ZnO@ED NPs.

Scanning electron microscopy was applied to examine the surface morphology of nanoparticles. Quality and efficacy are significantly influenced by the size of metallic NPs. As a result of their small dimensions and large surface area, ZnO NPs exhibit enhanced efficacy in killing microorganisms.^57^ The SEM analysis revealed a spherical structure of these ZnO@ED NPs with an average particle size of 57.94 ± 1.8 nanometers as depicted in Fig. 3(A and B) (histogram). The findings of the current study were in accordance with the average size of 50 nm reported by ref. 52.

### 3.6 Energy dispersive X-ray spectroscopy (EDS)

The confirmation of the presence of zinc metal was evaluated through the analysis of Energy Dispersive X-ray Spectroscopy (EDS) spectra. The spectra exhibited three distinct peaks in the energy range of 1 to 10 keV, with the most prominent peak observed at 1 keV as shown in Fig. 3(C)^21^. The stoichiometric ratio of oxygen to zinc was 9.86 : 83.24. The synthesized nanoparticles are capped by stabilizing compounds derived from the plant extract, which can explain the presence of carbon detected in the spectra. The EDS spectra have consistently shown that the peak of Zn is consistently detected in a similar region in several previous studies.^58,59^

### 3.7 Dynamic light scattering (DLS)

**Fig. 4:**
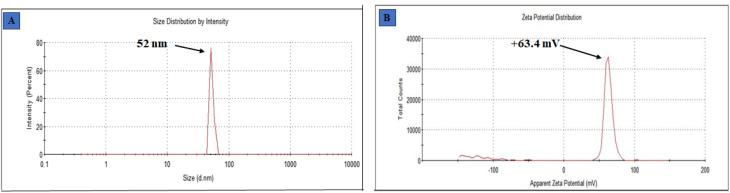
(A) Particle size, (B) zeta potential spectra of ZnO@ED NPs.

The determination of particle size was done by dynamic light scattering, using a Malvern Zetasizer Nano ZS instrument. Dynamic light scattering (DLS) is a commonly employed analytical technique utilized for the determination of particle size in colloidal solutions. Fig. 4(A) displays the size obtained from employing this methodology to measure the particle size of synthesized ZnO@ED NPs. The size of the green synthesized ZnO@ED nanoparticles ranges from 52 nm, (St Dev 3.416 nm). The fact that the DLS size is larger than the crystalline size as measured by XRD as seen in Fig. 2(A). When compared to XRD, DLS is a more efficient and cost-effective way to measure a large number of samples. DLS also gives a much bigger size, which could be because of the hydrodynamic shell. The shape and roughness of a particle are additional factors that potentially influence its hydrodynamic shell size.^60^ The physicochemical properties, like dimensions, shape, surface area, composition, and zeta potential of nanomaterials significantly impact their biological activities.^61^

### 3.8 Zeta potential

Zeta potential analysis was performed to quantify their surface charges in order to determine the ZnO NPs liquid stability. The measurement of the zeta potential yielded a value of +63.4 mV (St Dev 5.87 mV), as depicted in Fig. 4 (B). The charge that develops between 0 and ± 5 mV of zeta potential, coagulation occurs rapidly, whereas intermediate instability occurs between ±10 and ±30 mV, excellent stability occurs between ±40 and ±60 mV, and exceptional stability occurs above ±61 mV.^62^ The significant magnitude of this value suggests that the particles are actively resistant against each other, hence enhancing the stability of the nanoparticles.^63^

### 3.9 Photocatalytic degradation of methylene blue (MB)

**Fig. 5:**
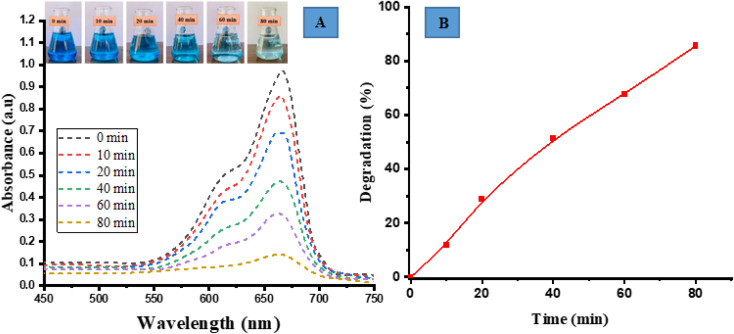
Photocatalytic degradation of methylene blue (MB) (A) spectrum degradation, (B) percentage degradation in the presence of sunlight.

The photocatalytic degradation activity of MB (a cationic dye) was performed by irradiating in sunlight. The degradation of the dye was observed over a period of 80 min by analyzing its UV-vis spectrum and visually examining the samples at specific time intervals of 0, 10, 20, 40, 60 and 80 min. Over a period of time and when exposed to direct sunlight, the original dark blue colour of MB gradually loses its intensity and transforms into a lighter shade. The spectrum degradation is shown in Fig. 5(A). The spectral observations indicated that the characteristic peak of MB at 663 nm exhibited a decaying trend following an exponential function, signifying the degradation of the dye.

The percent degradation of MB dye was determined by the following equation.^14^8
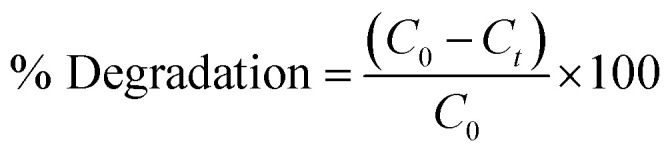


The graph depicted in Fig. 5(B) illustrates the correlation between the variable of time and the percentage of degradation. The presented figure illustrates a notable correlation between the degradation percentage and the duration of time. Specifically, it reveals that the ED@ZnO catalyst was able to degrade 85.61% of the MB over an 80 min period under the sunlight. Our finding aligns with the previously reported studies conducted by ref. 14 and 64.

### 3.10 Photocatalytic degradation of methyl orange (MO)

**Fig. 6:**
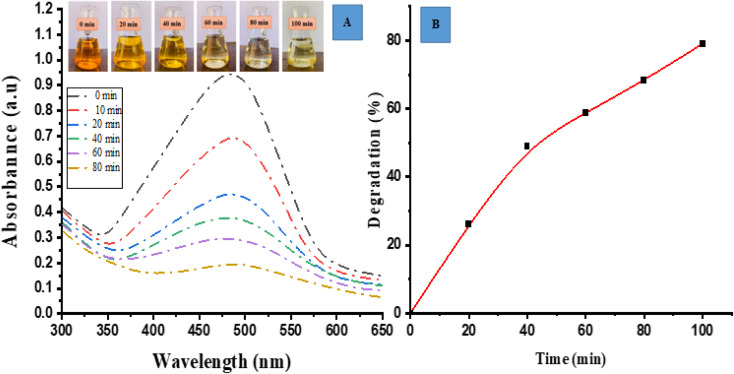
Photocatalytic degradation of methyl orange (MO), (A) spectrum degradation, (B) percentage degradation in the presence of sunlight.

Photocatalytic degradation activity for MO (anionic dye) by ED@ZnO NPs was carried out under sunlight. Visual observation and UV-vis spectroscopy were employed to check the degradation of dye across various time intervals (0, 10, 20, 40, 60, 80 min). Fig. 6(A) depicts the spectrum overlap observed during the gradual elimination of dye over a period of time. The spectral analysis showed a distinct peak at 465 nm, which exhibited an exponential decline, indicating the degradation of the dye.

The degradation percentage of MO dye was determined using the following eqn (9):9
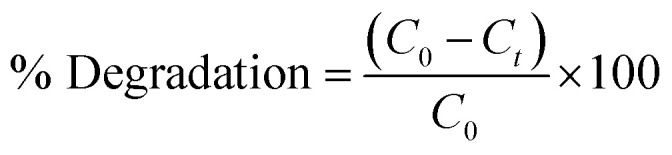


In Fig. 6(B), a graph is presented with the *Y* axis representing the percentage of degradation and the *X* axis representing the time. The data presented in this figure clearly demonstrates a progressive increase in the percentage of degradation over time. Specifically, the ED@ZnO NPs catalyst achieved a degradation rate of 79.10% for MO within an 80 min time span under the sunlight. Our finding is consistent with the earlier study conducted by.ref. 14

### 3.11 Degradation of 4-nitrophenol (PNP)

**Fig. 7:**
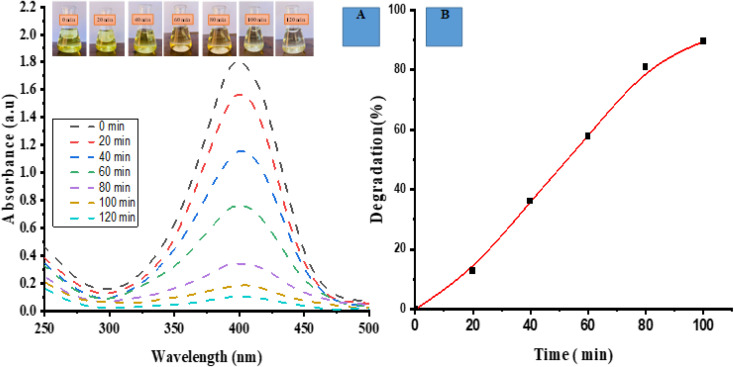
Photocatalytic degradation of 4-nitrophenol (PNP) (A) spectrum degradation, (B) percentage degradation in the presence of sunlight.

Under the presence of light, the ED@ZnO NPs exhibited photocatalytic degradation of the PNP dye. Recordings of the visual and UV-vis spectra were made at the time intervals of 0, 20, 40, 60, 80, 100 and 120 min in order to monitor the degradation of the dye. The spectrum shown in Fig. 7(A) illustrates the overlapping phenomenon observed throughout the process of dye removal. The spectral analysis revealed a gradual reduction in the intensity of the PNP peak (400 nm) as the dye underwent degradation, suggesting an exponential decay pattern.

The degradation percentage of PNP dye was determined using the following eqn (10):10
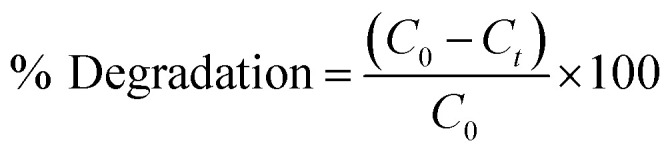


Fig. 7(B) illustrates the correlation between time and the percentage of degradation. The figure illustrates a consistent increase in the rate of degradation over time. Notably, the ED@ZnO catalyst was able to degrade 89.95% of the PNP within a 2 h period of exposure to sunlight, aligning with findings from previous research.^14^ The percentage degradations are similar to those found in the previous studies utilizing ZnO catalysts, as shown in Table 2.

**Table 2:** Comparison of photodegradation with previous literature

Source	Dyes	Irradiation	% Degradation	Irradiation time (min)	References
Biosynthesis/*Syzygium cumini*	MB	UV light	84	60	65
	MO		88	150	
Wild olive	MO	Natural sun	92.1	90	66
*Codonopsis lanceolata*	MB	UV light	90	40 min	64
*Moringa oleifera*	MO	Natural sun	70	180	67
*Equisetum diffusum* D	MB	Natural sun	85.61	80	This work
	MO		79.10	80	
	PNP		89.95	120	

### 3.12 Kinetics rate of photodegradation of MO, MB and PNP at ZnO NPs

**Fig. 8:**
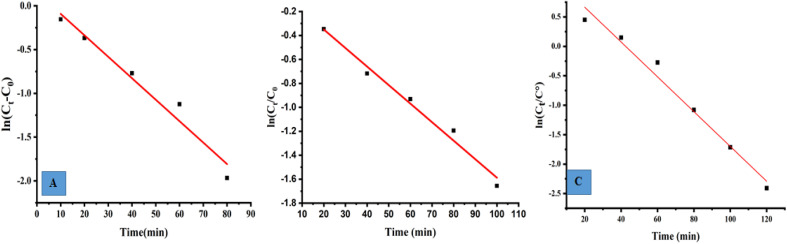
The photocatalytic reaction's kinetics study by plotting ln(*C*_*t*_/*C*_0_) against irradiation time for the first order reaction degradation, (A) methylene blue (MB), (B) methyl orange (MO) and (C) 4-nitrophenol (PNP).

The photocatalytic reaction's kinetics were studied by plotting ln(*C*_t_/*C*_0_) against irradiation time as illustrated in (Fig. 8A, MB), (8B, MO) and (8C, PNP) respectively. The resulting linear relationship was described by the following eqn (11):11
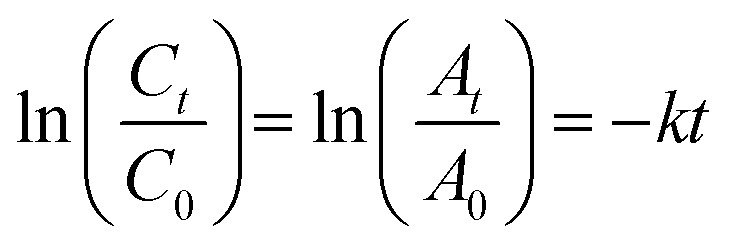
where *C*_0_ is the concentration reaction mixture at zero reaction time and *C*_*t*_ is the concentration at time interval *t*. The slope of the linear fit was applied to calculate the rate constant, which is represented by *k*. All three dyes (MB, MO and PNP) exhibited a reasonable degree of linearity, suggesting that the photodegradation process underwent first-order reaction kinetics. While the intercept value for MB is 0.152 ± 0.131 and with an *R*^2^ value of 0.953, the intercept value for MO is −0.040 ± 0.077, with a correlation coefficient *R*^2^ of 0.977 and the intercept value for PNP is 1.256 ± 0.164, with a correlation coefficient *R*^2^ of 0.974.

### 3.13 Mechanism of photodegradation

There are three kinds of semiconductor heterojunction arrangements depending on band gap energies.^68^ In straddling gap structure (type 1), semiconductor 2 has a greater negative capacitance (CB) and a greater positive valence (VB) than semiconductor 1. Electrons and holes will accumulate in semiconductor 1 at a reduced *E*_g_, in accordance with the charge carrier transfer principle. Due to a smaller *E*_g_, charge carrier recombination might occur, resulting in a decrease in photocatalytic efficiency. In a staggered gap configuration (type 2), the CB and VB of semiconductor 2 are more negatively associated with each other than those of semiconductor 1. As a result, holes will go from semiconductor 1 to 2, and electrons will travel from semiconductor 2 to 1. Type 2, in contrast to type 1, provides better electron–hole separation as a result of the compartmentalization of charge carriers into two semiconductors. In the third broken gap construction type, semiconductor 2 outperforms semiconductor 1 in terms of CB and VB. Therefore, electrons and holes are unable to enter their respective bands due to the interface of the semiconductor. The energy barrier at the interface prevents charge carriers from passing across, causing this to happen.^69^ Of the three types of semiconductor heterojunction structures, type 2 is by far the most prevalent. It improves the photocatalytic process by facilitating the efficient separation of photo-generated electrons and preventing charge carrier recombination during electron transfer.^70^

**Fig. 9:**
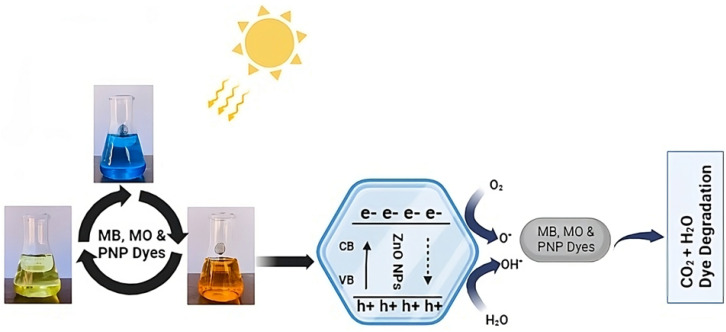
Possible mechanism of photodegradation of MB, MO and PNP dyes.

Dye-based model molecules for semiconductor photocatalytic activity may interact with degradation intermediates that absorb at the dye's wavelength.^71^ Photocatalysis, dye sensitization, or both might lead to the degradation of sensitive dyes.^72^ Thus, distinguishing between the two effects is essential for evaluating the synthesized catalyst's activity and understanding the degradation process. The photocatalytic degradation process of MB, MO and PNP at ZnO NPs is shown in Fig. 9, which is based on prior research and our results.^14,72^ The following mechanism usually takes place during the photocatalytic degradation of ZnO NPs:ZnO + *hν* → ZnO + e^−^ + h^+^O_2_ + e^−^ → O_2_˙^−^h^+^ + H_2_O → OH˙ + H^+^OH˙ + O_2_˙^−^ + organic dye (MB/MO/PNP) → mineral acids + CO_2_ + H_2_O

### 3.14 Antibacterial activity

**Fig. 10:**
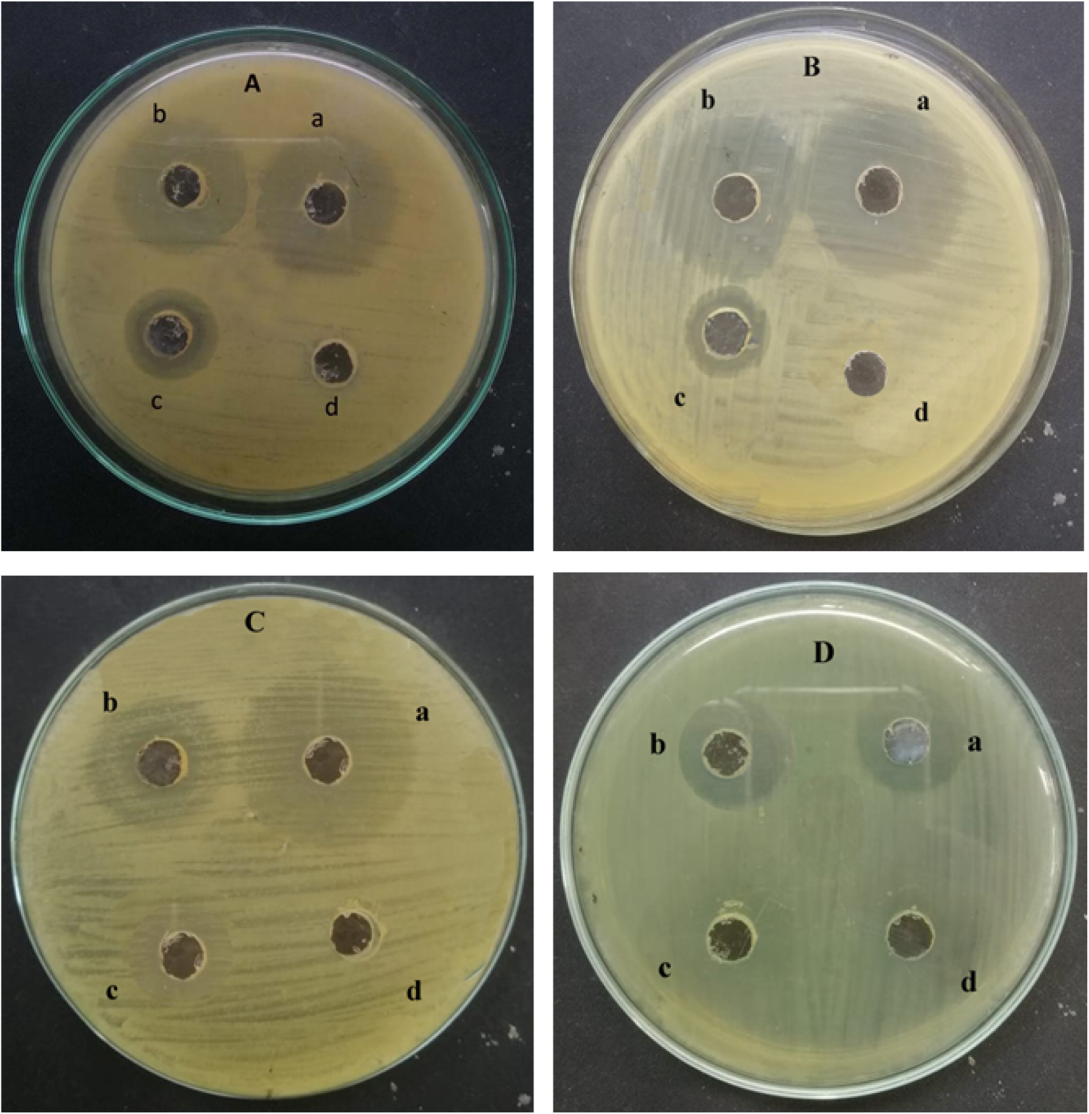
Antibacterial activity of the green synthesised ED@ZnO NPs (A) *Listeria monocytogenes* (ATCC#13932) (a) positive control (17 mm), (b) ED@ZnO NPs (16 mm), (c) plant extract (8 mm), (d) negative control DH2O (0 mm), (B) *Staphylococcus epidermidis* (ATCC#12228) (a) positive control (19 mm), (b) ED@ZnO NPs (18 mm), (c) plant extract (8 mm), (d) negative control DH2O (0 mm), (C) *Escherichia coli* (ATCC#10536) (a) positive control (19 mm), (b) ED@ZnO NPs (14 mm), (c) plant extract (6 mm), (d) negative control DH2O (0 mm), (D) *Bordetella bronchiseptica* (ATCC#4617) (a) positive control (13 mm), (b) ED@ZnO NPs (11 mm), (c) plant extract (4 mm), (d) negative control DH2O (0 mm).

**Fig. 11:**
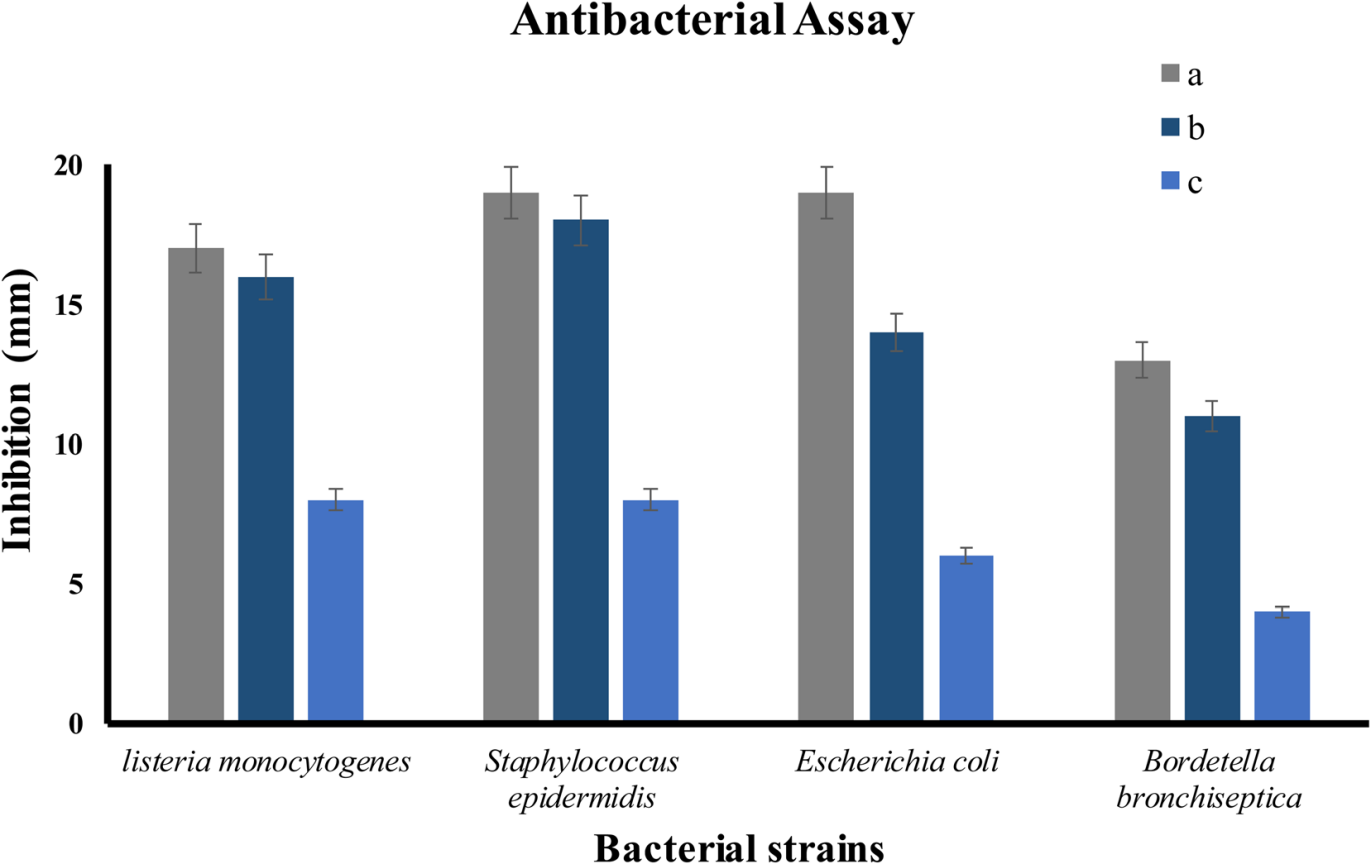
The percentage inhibition graph, (a) positive control, (b) ED@ZnO NPs and (c) plant extract.

The antibacterial activity of ED@ZnO NPs was examined using both Gram-positive *L. monocytogenes* (ATCC#13932) and *S. epidermidis* (ATCC#12228), as well as Gram-negative *E. coli* (ATCC#10536) and *B. bronchiseptica* (ATCC#4617). The well diffusion technique was used to quantify the zones of inhibition (ZOI) exhibited by Gram-positive and Gram-negative bacteria as shown in Fig. 10 and the graphical representation in Fig. 11. The inhibitory effects of ED@ZnO NPs on *L. monocytogenes* (ATCC#13932), (16 mm), *S. epidermidis* (ATCC#12228), (18 mm), *E. coli* (ATCC#10536), (14 mm) and *B. bronchiseptica* (ATCC#4617), (11 mm). It was observed that these bacterial strains exhibited various degrees of inhibition. In comparison to Gram positive bacteria, Gram negative bacteria displayed reduced susceptibility to ED@ZnO NPs NPs.^73^

ZnO NPs exhibit the capability to attach to both Gram-positive and Gram-negative bacteria surfaces *via* different pathways. Cell surface negative charges are introduced by teichoic acid in the peptidoglycan layer and lipoteichoic acid in the membrane. Electrostatic interactions bring positive charges from ZnO NPs to the surface of the cell. The difference in electrostatic gradients causes damage to the cell surface.^73,74^ Zn^2+^ ions are chelated by teichoic and lipoteichoic acids and then transferred across membrane proteins *via* passive diffusion. Additionally, the bactericidal action may take place through different mechanisms *i.e.*, adsorption on the bacterial surface, electrostatic interactions and the formation of different intermediates. Hydrogen ion passage across the cell membrane allows metallic ions to diffuse, establishing the electrochemical gradient.^75^ Particle size plays a significant role in this process because electrostatic interactions are stronger between smaller particles. Zinc oxide inhibitory effect is therefore dependent on parameters including size, concentration, and contact length.^76^

In this study, the broth dilution assay was used to determine the MICs against *Listeria monocytogenes* (ATCC#13932), *Staphylococcus epidermidis* (ATCC#12228), *Escherichia coli* (ATCC#10536) and *Bordetella bronchiseptica* (ATCC#4617). The minimum inhibitory concentration (MIC) values are shown in Table 3. The results showed that the MIC of ED@ZnO NPs were 30 and 20 μg mL^−1^ for *Listeria monocytogenes* (ATCC#13932), *Staphylococcus epidermidis* (ATCC#12228), and 70, 90 μg mL^−1^ for *Escherichia coli* (ATCC#10536) and *Bordetella bronchiseptica* (ATCC#4617). This suggests that the synthesized ED@ZnO NPs effectively inhibited the growth of these 4 pathogens at relatively low concentrations. The current MIC data supports previous results by showing that the Gram-negative bacterial strain *Escherichia coli* (ATCC#10536) and *Bordetella bronchiseptica* (ATCC#4617) are more resistant to ED@ZnO NPs than the Gram-positive bacterial strains *Listeria monocytogenes* (ATCC#13932), *Staphylococcus epidermidis* (ATCC#12228).^77^ This may be due to variations in their cell walls composition or lipopolysaccharides blocking ED@ZnO NPs positive charges, reducing *Escherichia coli* (ATCC#10536) and *Bordetella bronchiseptica* susceptibility to ED@ZnO NPs. The results show that ED@ZnO NPs have a considerable antibacterial effect on the investigated microbes. The enhanced antibacterial activity is attributed to the bioactive compounds present in the extract that cape the nanoparticles, as well as the larger surface area of the nanoparticles.

**Table 3:** Zone of inhibition (mm) and MIC of ED@ZnO NPs

Bacterial strains	Zone of inhibition (mm)	MIC (μg mL^−1^)
*Listeria monocytogenes*	16	30
*Staphylococcus epidermidis*	18	20
*Escherichia coli*	14	70
*Bordetella bronchiseptica*	11	90

### 3.15 Antioxidant activity

**Fig. 12:**
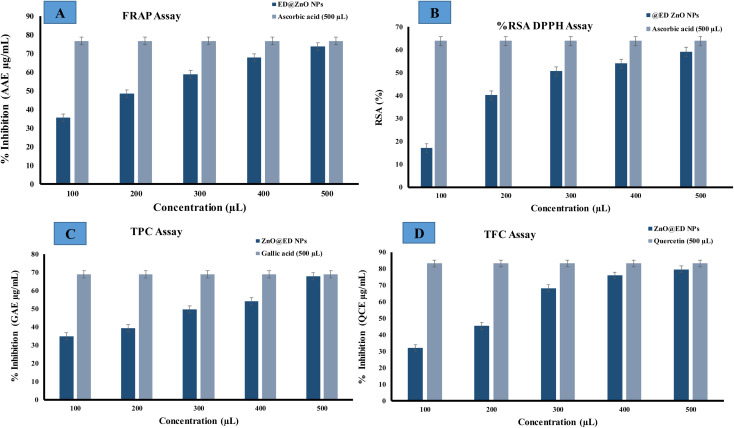
Antioxidant activity of the green synthesized ED@ZnO NPs, (A) ferric reducing antioxidant assay (FRAP), (B) 2,2-diphenyl-1-picrylhydrazyl (DPPH) radical, (C) total phenolic content (TPC) and (D) total flavonoid content (TFC).

Total antioxidant activity was estimated with the ferric reducing antioxidant assay (FRAP). Ascorbic acid is commonly utilized due to its role as a secondary antioxidant, whereby it effectively counteracts the harmful effects of free radicals and interrupts chain reactions. The efficacy of ascorbic acid as an antioxidant can be attributed to its substantial polyhydroxyl content and the presence of free hydroxyl groups, which effectively counter harmful free radicals.^36^ In this study, a solution of trichloroacetic acid (TCA) was employed for the purpose of eliminating the deposited potassium ferrocyanide (K_3_Fe(CN)_6_). Upon the addition of FeCl_3_, a complex is formed which show a chromatic range from green to blue. The reducing power of greenly synthesized ED@ZnO NPs was assessed by measuring their antioxidant activity by converting Fe^+3^ to Fe^+2^. Radical scavenging activity exhibited a range of 35.55 μg (AAE μg mL^−1^) to 73.73 μg (AAE μg mL^−1^) across the concentration range of 100 to 500 μL, measured against an ascorbic acid standard curve, as illustrated in Fig. 12(A). The antioxidant activity of the synthesized nanoparticles is enhanced by mechanisms such as simple electron transfers, sequential proton loss and electron transfer, proton transfer or the transfer of hydrogen atoms, which stabilize the radicals.^36^

The capacity of ZnO NPs to eliminate DPPH (free radicals) was assessed by a DPPH assay, encompassing doses ranging from 100 to 500 μL. The efficacy of ZnO NPs was evaluated in comparison to the conventional antioxidant ascorbic acid in terms of their capacity to suppress the DPPH radical. As the concentration of ZnO NPs increased, there was an observed enhancement in their capability to inhibit the generation of the 2,2-diphenyl-1-picrylhydrazyl (DPPH) radical. The DPPH assay serves as a reliable indicator for determining the antioxidant properties of a given chemical compound. This suggests that the existence of bioactive chemicals in the extract may effect this process. The scavenging capacity of ZnO NPs (100 to 500 μL) against DPPH was assessed, and the results are presented in Fig. 12(B). The range of DPPH radical suppression percentages exhibited by ZnO NPs varied from (17.03, 40.14, 50.62, 53.93, 59.17 μg) (AAE μg mL^−1^) for ED@ZnO NPs concentrations ranging from 100 to 500 μL.

The total phenolic content (TPC) and total flavonoid content (TFC) have also been evaluated, by establishing a standard curve with gallic acid (GA) and quercetin (QC) based on the absorbance–concentration relation are shown in (Fig. 12C and D). The study revealed that there were concentration-dependent elevations in the total phenolic content (TPC) and total flavonoid content (TFC), with recorded values ranging from 34.78, 39.36, 49.59, 54.15, 67.94 μg) (GAE μg mL^−1^) and (32.09 45.36 68.31 76.04 79.59, μg (QCE μg mL^−1^) respectively. The observed results suggest a potential antioxidant effect, which can be attributed to the bioactive constituents that aid in the reduction of metal ions, leading to the creation of nanoparticles.^36,76^

### 3.16 Anti-inflammatory activity

There is a general belief that medicinal plants possess a considerable quantity of novel chemicals that may confer therapeutic benefits. Hence, it is logical to consider plants as a prospective reservoir of innovative anti-inflammatory pharmaceuticals to complement conventional treatments. Inflammation can lead to chronic organ damage and potentially fatal hypersensitivity responses.^78^ NSAIDs are believed to inhibit the denaturation of proteins antigens, potentially mitigating autoimmune diseases. Protein denaturation is a recognized stimulus for the inflammatory response of body in inflammatory disorders like rheumatoid arthritis.^79^ The anti-inflammatory effects of ED@ZnO NPs may be attributed to their ability to prevent protein denaturation. The observed anti-inflammatory effect of ED@ZnO NPs was dosage dependent, as shown in Table 4. The highest concentration of synthesized ED@ZnO NPs at 500 μL (1 mg mL^−1^) exhibited a percentage of 91.17%, while the lowest concentration at 100 μL (1 mg mL^−1^) shown a percentage of 23.70%. The current research discovered that the secondary metabolites found in the plant extract of *Equisetum diffusum* D effectively served as capping agents for the ED@ZnO NPs. Previous studies have demonstrated that secondary metabolites derived from plant extracts has the ability to impede the release of neutrophil lysosomal components at inflammation site induced by ED@ZnO NPs. The release of lysosomal proteinases and bactericidal enzymes into the extracellular space has been found to contribute to the exacerbation of inflammation and tissue damage.^80^

**Table 4:** Anti-inflammatory activity of the synthesized ED@ZnO NPs

Concentration (μL)	ED@ZnO NPs (% inhibition)	Diclofenac sodium (500 μL) (% inhibition)
100	23.70	
200	60.99	
300	83.64	94.30
400	87.76	
500	91.17	

## 5 Conclusion

Herein we demonstrated a green approach for the synthesis of ZnO NPs using an aqueous extract of *Equisetum diffusum* D, with its essential phyto-constituents, such as phenols and flavonoids, which facilitated the stabilization and reduction of the nanoparticles. The UV-vis absorption spectroscopy confirmed the synthesis of ZnO NPs with a consistent pale yellow color and a characteristic absorbance peak at 371 nm due to surface plasmon resonance. X-ray diffraction analysis shows a hexagonal wurtzite crystal structure, and the average crystallite size of approximately 18.84 nm was determined. Fourier transform infrared spectroscopy supported the role of phytochemicals in stabilizing and reducing the nanoparticles. Scanning electron microscopy depicted spherical structures with an average particle size of 57.94 nm. Energy Dispersive X-ray Spectroscopy confirmed the presence of zinc in the synthesized nanoparticles. Dynamic light scattering revealed a particle size of 52 nm, and the zeta potential indicated good stability. The synthesized ZnO NPs exhibited significant photocatalytic degradation of methylene blue, methyl orange, and *p*-nitrophenol under sunlight exposure. Additionally, the nanoparticles demonstrated selective colorimetric detection of Hg^2+^ ions and displayed antimicrobial activity to both Gram-positive and Gram-negative bacteria. The nanoparticles exhibited notable antioxidant activity, scavenging DPPH radicals, and reducing ferric ions. Furthermore, the anti-inflammatory potential of the synthesized ZnO NPs, due to their ability to inhibit protein denaturation, was dose-dependent. Overall, the green-synthesized ZnO NPs using *Equisetum diffusum* D extract exhibited multifaceted properties, including photocatalytic, antimicrobial, antioxidant, and anti-inflammatory activities, showcasing their potential for various applications in environmental and biomedical fields. Especially, these nanoparticles may serve as safe and nontoxic materials for the purification of industrial and other waste waters. Industrial effluents contaminate the environment, especially water bodies, with pollutants, including dyes having high COD, BOD, and therefore should be removed from the effluents. Methylene blue, methyl orange and 4-nitrophenol are commonly used dyes in paper, textile and leather industries for dying various products, but pose serious concerns for the public health and environment. These ZnO NPs are able to purify the dyes contaminated water efficiently and therefore applicable in the water treatment, including the industrial waste water/effluents.

## Data availability

The data are available upon reasonable request.

## Author contributions

NA: methodolgy, data curation, resources, writing – original draft; AA: methodology, conceptualization, writing – review & editing; MFR: supervision, formal analysis, visualization, resources; MN: supervision, conceptualization, funding acquisition, writing – review & editing.

## Conflicts of interest

There are no conflicts to declare.
